# Causal relationship between immunophenotypes and mitral valve prolapse: a bidirectional Mendelian randomization study

**DOI:** 10.3389/fcvm.2024.1404284

**Published:** 2024-10-03

**Authors:** Yue Wang, Yusi Shen, Lina Tan, Liangbo Hu, Min He, Xiaocong Zeng

**Affiliations:** ^1^ Department of Cardiology, The First Affiliated Hospital of Guangxi Medical University, Nanning, Guangxi, China; ^2^ Second Department of Orthopedic Rehabilitation, Taihe Hospital, Hubei University of Medicine, Shiyan, China; ^3^ Guangxi Key Laboratory Base of Precision Medicine in Cardio-Cerebrovascular Diseases Control and Prevention & Guangxi Clinical Research Center for Cardio-Cerebrovascular Diseases, Nanning, Guangxi, China; ^4^ School of Basic Medical Sciences, Guangxi Medical University, Nanning, Guangxi, China

**Keywords:** Mendelian randomization analysis, mitral valve prolapse, genetic approaches, immune cells, immunophenotypes

## Abstract

**Background:**

Emerging evidence indicates a significant link between various immune cell types and the development of heart valve disorders. Mitral valve prolapse (MVP) is a common condition that can lead to heart failure, arrhythmias, and even sudden death. Currently, the role of immune cells in MVP is not well understood. Thus, this study aimed to explore the causal relationship between immunophenotypes and the risk of MVP.

**Methods:**

This study conducted a two-sample Mendelian randomization (MR) analysis to examine the link between 731 immunophenotypes and MVP. Publicly available data from genome-wide association studies were used for both the exposures and outcomes. The primary method for assessing the causal relationship between mitral valve prolapse and the 731 immunophenotypes was the inverse variance weighted method. Additionally, to ensure the MR results were reliable and valid, sensitivity analyses, including leave-one-out analysis, the Cochran *Q*-test, and the Egger intercept test, were conducted.

**Results:**

The findings indicated that multiple immune cell phenotypes potentially cause changes in the risk of developing MVP. After adjusting for the false discovery rate, nine immune phenotypes were found to increase the risk of MVP, while nine others appeared to decrease it. In addition, reverse MR analysis found no causal relationship between MVP and these eighteen immunophenotypes.

**Conclusion:**

Through genetic analyses, this research demonstrated a significant causal relationship between certain immune cells and MVP, providing new insights for future basic and clinical research.

## 1 Introduction

Mitral valve prolapse (MVP) is the most common form of valvular heart disease, affecting approximately 2%–3% of the general population in Western countries (1, 2). It occurs when one or both mitral valve leaflets bulge into the left atrium by at least 2 mm above the mitral annulus during systole, as observed in an echocardiogram (3, 4). Although often benign, MVP can lead to severe complications such as significant mitral regurgitation and heart failure and is a primary cause of valve repair surgery. In some cases, it may cause life-threatening ventricular arrhythmias and sudden cardiac death, alongside severe mitral regurgitation and left ventricular dysfunction (5, 6). Presently, because of a limited understanding of the mechanisms underlying MVP, only a few known preventive or recommended treatment strategies are available currently, leaving surgery as the only option once the disease advances.

For decades, clinicians have learned from infectious diseases and the immune activation that affects heart function (7, 8). Immunotherapy for cardiovascular diseases has emerged as a significant focus within the scientific community. In mouse models of myocardial infarction, transverse aortic constriction, and hypertension, mice with adoptive T cell transfers that express a chimeric antigen receptor against fibroblast activation protein showed a marked decrease in cardiac fibrosis and a restoration of function (9). Delivery of therapeutic mRNA into the body via lipid nanoparticles to form transient anti-fibrotic chimeric antigen receptor T cells also improved cardiac function in mouse models of heart failure (10). Research highlights the critical role of the immune system not only in cardiac repair but also in MVP development (11). The two primary causes identified are myxomatous MVP and fibroelastic deficiency, with various cytokines and chemokines playing a role in the disease (12). By utilizing a mouse model of Marfan syndrome (MFS) defined by a mutation in the Fibrillin-1 (FBN1) gene, researchers have discovered a connection between mitral valve pathology and the enhanced activity of transforming growth factor-*β* (TGF-*β*) (13), which plays a significant fibrotic role in MVP development. Treatment with angiotensin II blockers has been shown to reduce TGF-*β* activity and mitigate MVP by affecting extracellular matrix production (13, 14). MFS mice with myxomatous valve degeneration showed a notable increase in infiltrating cells expressing MHCII and CCR2, along with increased chemokine activity and changes in the inflammatory extracellular matrix were noted (15), correlating with findings in human and mouse tissues of myxomatous disease (16). A notable link between immune response and MVP was also observed in patients with inflammatory bowel disease (IBD), who had a high incidence of MVP and mitral leaflet thickening in patients with IBD (17). These findings suggest that abnormal immune responses and inflammation may be key factors in MVP pathogenesis. However, current research is limited by small sample sizes, study design issues, potential reverse causality, and unaddressed confounding factors.

Mendelian randomization (MR) is an analytical technique founded on the principle of Mendelian inheritance, predominantly used in the inference of epidemiological etiology (18). This method employs genetic variants, usually single-nucleotide polymorphisms (SNPs), as proxies for clinical interventions to assess their association with outcomes following exposure (19). MR thus enables causal inference, effectively bridging the divide between observational epidemiology and interventional trials (20). It is critical for the causal sequence in MR to be logical, as an individual's genetic code is fixed at birth. A detailed two-sample MR analysis was conducted in our study to establish the causal link between immune cell signatures and MVP.

## 2 Materials and methods

### 2.1 Study design

**Figure 1:**
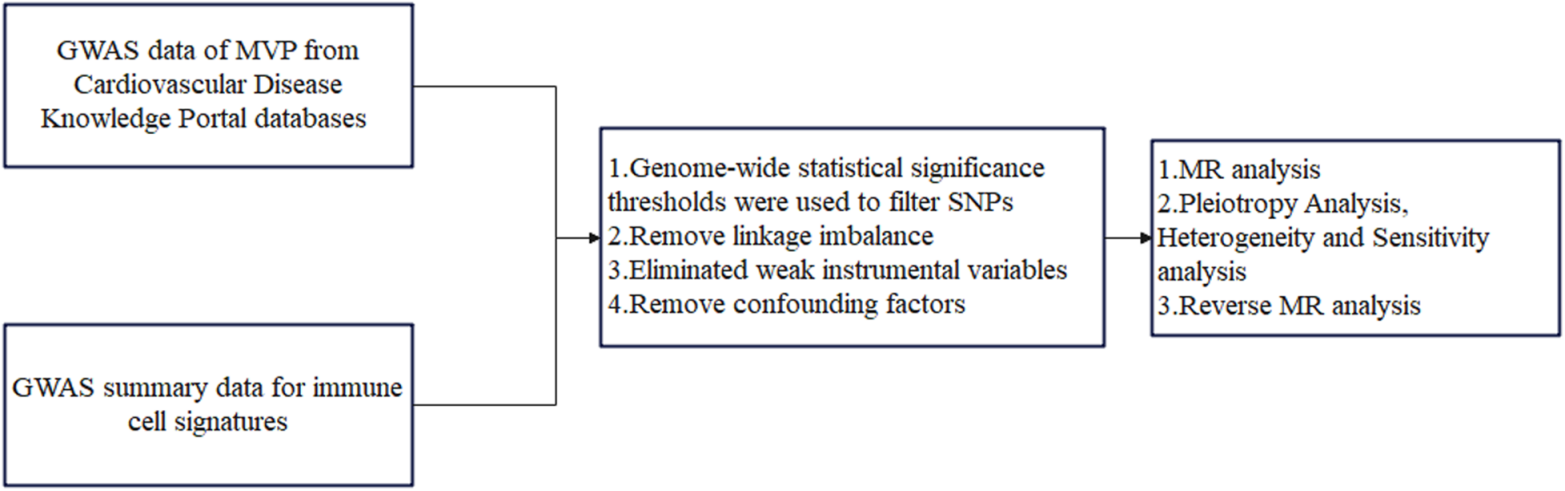
The flowchart of the study. MVP, mitral valve prolapse; SNPs, single-nucleotide polymorphisms; MR, Mendelian randomization.

This study conducted a two-sample MR analysis to explore the potential causal relationship between 731 immune cell signatures (across 7 panels) and MVP. Our study design is illustrated in Figure 1. MR utilizes genetic variations to estimate the effect of exposure, relying on three critical assumptions for valid instrumental variables (IVs): (1) the genetic variation is strongly associated with the exposure; (2) the genetic variants are not associated with any confounders of the exposure-outcome relationship; (3) the genetic variation affects the outcome solely through the exposure. This study was exempt from further ethical approval or informed consent due to the use of publicly available data (21).

### 2.2 Genome-wide association study (GWAS) summary data for MVP

Data for MVP were extracted from genome-wide association studies (GWAS) from the Cardiovascular Disease Knowledge Portal databases (https://cvd.hugeamp.org/). The data included contributions from the Framingham Heart Study (FHS), the Mass General Brigham (MGB) Biobank, the UK Biobank, the Broad Cardiovascular Disease Initiative (Broad CVDi), the MVP-FRANCE/Three-City (3C), and the MVP-NANTES/D.E.S.I.R. The GWAS involved a total of 439,533 individuals from European and American populations (4,884 cases and 434,649 controls) and approximately 7.5 million SNPs (22), leading to the identification of 14 significant (*P* < 5 × 10^−8^) SNPs associated with MVP.

### 2.3 Immunity-wide GWAS data sources

The GWAS Catalog (accession numbers: GCST0001391 to GCST0002121) provides public access to summary statistics for immune traits (23), encompassing a combined total of 731 immunophenotypes. These phenotypes include absolute cell counts (AC, *n* = 118), median fluorescence intensity (MFI, *n* = 389) for surface antigen levels (*n* = 389), morphological parameters (MP, *n* = 32), and relative cell counts (RC, *n* = 192). These measurements span various cell types and stages, including B cells, conventional dendritic cells (DCs), T-cell maturation stages, monocytes, myeloid cells, TBNK (T cells, B cells, natural killer cells), and Treg panels, with MPs focusing on cDC and TBNK panels. The immunophenotype GWAS, involving 3,757 Europeans from non-overlapping cohorts, accounted for sex, age, and the square of age as covariates. Approximately 22 million SNPs were analyzed using high-density arrays and imputed with a reference panel based on the Sardinian genome (24).

### 2.4 Selection of instrumental variables (IVs)

In the research (23, 25, 26), IVs were selected based on variants with *P* < 1 × 10^−5^. These SNPs were then pruned using the clumping procedure in PLINK software (version 1.90), setting the linkage disequilibrium (LD) *r*^2^ threshold to below 0.1 within a 500 kb distance. The 1,000 Genomes Projects were used as a reference panel for calculating LD *r*^2^. The proportion of phenotypic variation was explained, and the F statistic was calculated for each IV to evaluate IV strength and avoid weak instrumental bias. The equation R2 was computed using R2 = [2 × EAF (1- EAF) × *β*2], with EAF representing the effect allele frequency and *β* representing the estimated genetic influence on immunophenotypes. To assess the strength of each SNP, we calculated the F-statistic using the following equation: F = [R2 × (n−1−k)]/[(1−R2) × k]. In this equation, R2 measures how much of the phenotypic variation can be attributed to the genetic variants, k represents the total number of SNPs, and n is the sample size (27). To prevent bias from weak independent variables (IVs), only IVs with an *F*-value greater than 10 were retained (28). Fourteen identified specific loci were used as instrumental variables for reverse Mendelian randomization of MVP and immunophenotypes (22).

### 2.5 Statistical analysis

All analyses were conducted using R software version 4.3.2 (http://www.Rproject.org). The causal relationship between 731 immunophenotypes and MVP was assessed using the “TwoSampleMR” package (version 0.5.8), employing methods such as inverse variance weighted (IVW), weighted median (WM), simple mode, weighted mode, and MR Egger. The IVW method was the primary estimator for causal effects in MR analysis (29). Scatter plots confirmed that outliers did not skew the results. Given the risk of type 1 errors from multiplex testing, false discovery rate (FDR) correction was applied. To evaluate heterogeneity among chosen variables, leave-one-out analysis, funnel plots, Cochran's Q statistic, and their respective *p*-values were employed. The Egger intercept was used to identify the influence of horizontal pleiotropy, indicating horizontal multiplicity when statistically significant.

## 3 Results

### 3.1 Exploration of the causal effect of immunophenotypes on MVP

**Figure 2:**
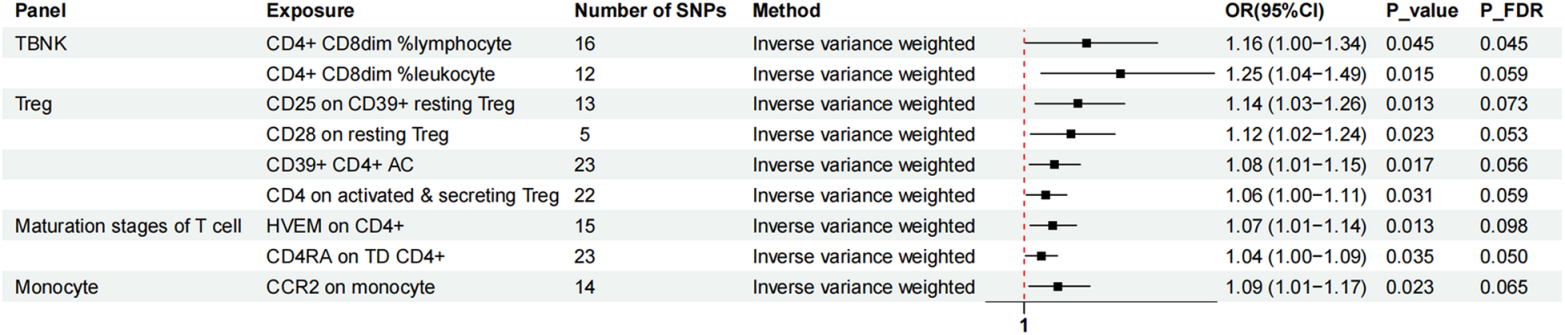
The forest plot showed the causal relationship in which nine immunophenotypes promoted MVP development by the IVW method. TBNK, T cells, B cells, Natural killer cells; AC, absolute cell; TD, terminally differentiated.

**Figure 3:**
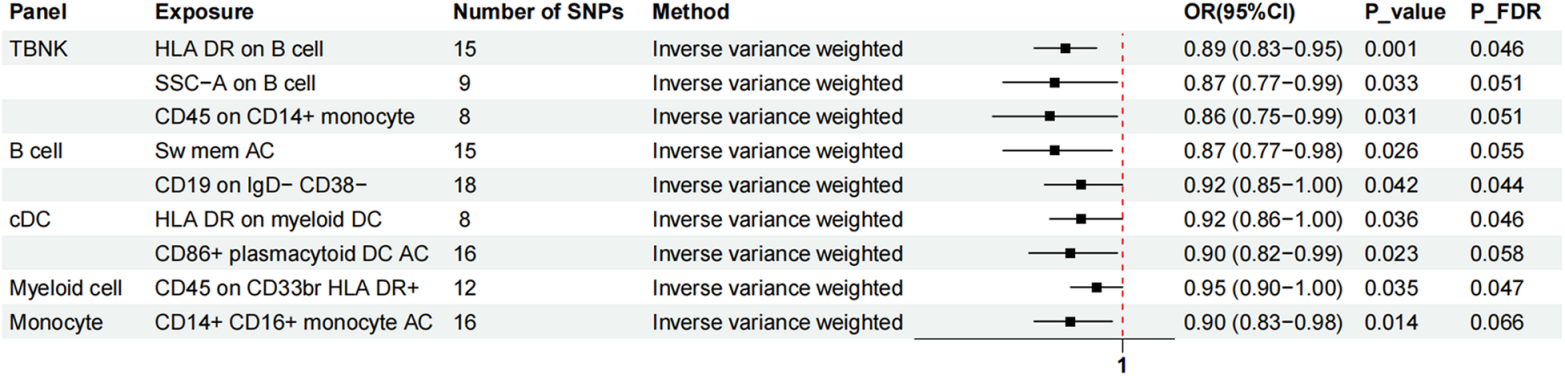
The forest plot showed the causal relationship in which nine additional immunophenotypes protected the development of MVP by the IVW method. TBNK, T cells, B cells, natural killer cells; AC, absolute cell; Sw mem, switched memory; DC, dendritic cell; HLA, human leucocyte antigen; br, bright.

Two-sample MR analysis was conducted to investigate the causal effect of immunophenotypes on MVP. To correct for multiple comparisons, the FDR method was applied, setting the significance threshold at an FDR-adjusted *P*-value (P_FDR_) of 0.1. The study identified eighteen immunophenotypes across various cell panels, including five from the TBKN panel, four from the Treg panel, and two each from the B cell, T-cell maturation stages, DC panel, and monocyte cell panels, with one from the myeloid cell panel. According to Table 1, comprehensive GWAS details on eighteen immunophenotypes can be obtained. The involvement of immune cells in MVP development, as demonstrated through the IVW method, is illustrated in Figures 2, 3.

**Table 1:** The GWAS information of eighteen immunophenotypes.

Panel	GWAS ID	Trait	Sanmple size	Number of SNPs	Trait type
TBNK	ebi-a-GCST90002116	HLA DR on B cell	3060	14891221	MFI
TBNK	ebi-a-GCST90002075	SSC-A on B cell	3112	14903706	Morphological parameter
TBNK	ebi-a-GCST90001909	CD45 on CD14 + monocyte	3112	14903652	MFI
TBNK	ebi-a-GCST90001610	CD4+ CD8dim%lymphocyte	3668	15198002	Relative count
TBNK	ebi-a-GCST90001611	CD4+ CD8dim%leukocyte	3668	15198002	Relative count
Treg	ebi-a-GCST90001938	CD25 on CD39 + resting Treg	3118	15067285	MFI
Treg	ebi-a-GCST90001900	CD28 on resting Treg	2919	4824972	MFI
Treg	ebi-a-GCST90001660	CD39+ CD4+ AC	3408	15135292	Absolute count
Treg	ebi-a-GCST90002070	CD4 on activated & secreting Treg	2920	14849646	MFI
Maturation stages of T cell	ebi-a-GCST90001875	HVEM on CD4+	1247	13730810	MFI
Maturation stages of T cell	ebi-a-GCST90002099	CD4RA on TD CD4+s	2903	14842283	MFI
B cell	ebi-a-GCST90001403	Sw mem AC	3656	15048937	Absolute count
B cell	ebi-a-GCST90001733	CD19 on IgD- CD38-	3657	15048951	MFI
Monocyte	ebi-a-GCST90001580	CD14+ CD16 + monocyte AC	3629	15038157	Absolute count
Monocyte	ebi-a-GCST90002008	CCR2 on monocyte	3629	15034296	MFI
cDC	ebi-a-GCST90002104	HLA DR on myeloid DC	2872	14829776	MFI
cDC	ebi-a-GCST90001466	CD86 + plasmacytoid DC AC	3374	15130102	Absolute count
Myeloid cell	ebi-a-GCST90002053	CD45 on CD33br HLA DR +	1580	14129891	MFI

SNPs, single-nucleotide polymorphisms; IVW, inverse variance weighted. TBNK, T cells, B cells, natural killer cells; AC, absolute cell; Sw mem, switched memory; DC, dendritic cell; HLA, human leucocyte antigen; TD, terminally differentiated; br, bright.

Nine immunophenotypes were associated with increased MVP risk: CD4^+^ CD8^dim^% lymphocytes, CD4^+^ CD8^dim^% leukocytes, CCR2 on monocytes, CD25 on CD39^+^ resting Treg cells, CD28 on resting Treg cells, CD39^+^ CD4^+^ AC, CD4 on activated and secreting Treg cells, herpesvirus entry mediator (HVEM) on CD4^+^ cells, and CD4 RA on TD CD4^+^ cells (Figure 2).

The study utilized the IVW method to estimate the odds ratios (ORs) for various immunophenotypes and their effect on the risk of MVP. A significant association was observed for CD4^+^ CD8^dim^% lymphocytes, with an OR of 1.16 [95% confidence interval (CI) = 1.00–1.34, *P* = 0.045, P_FDR_ = 0.045]. Similarly, CD4^+^ CD8^dim^% leukocytes showed an OR of 1.25 (95% CI = 1.04–1.49, *P* = 0.015, P_FDR_ = 0.059). CCR2 expression on monocytes was associated with an OR of 1.09 (95% CI = 1.01–1.17, *P* = 0.023, P_FDR_ = 0.065), and simple mode analysis provided consistent results (OR = 1.24, 95% CI = 1.05–1.47, *P* = 0.028). CD25 expression on CD39^+^ resting Treg cells was linked to an OR of 1.14 (95% CI = 1.03–1.26, *P* = 0.013, P_FDR_ = 0.073), with MR Egger analysis indicating an OR of 1.33 (95% CI = 1.12–1.58, *P* = 0.008). The influence of CD28 on resting Treg cells resulted in an OR of 1.12 (95% CI = 1.02–1.24, *P* = 0.023, P_FDR_ = 0.053). The OR for CD39^+^ CD4 ^+^ AC was 1.08 (95% CI = 1.01–1.15, *P* = 0.017, P_FDR_ = 0.056), with both MR Egger (OR = 1.12, 95% CI = 1.01–1.23, *P* = 0.038) and WM (OR = 1.08, 95% CI = 1.00–1.16, *P* = 0.039) analyses supporting these findings. The OR for CD4 on activated and secreting Treg cells was 1.06 (95% CI = 1.00–1.11, *P* = 0.031, P_FDR_ = 0.059). HVEM expression on CD4^+^ cells showed an OR of 1.07 (95% CI = 1.01–1.14, *P* = 0.013, P_FDR_ = 0.098), confirmed by MR Egger (OR = 1.16, 95% CI = 1.04–1.30, *P* = 0.018) and WM (OR = 1.12, 95% CI = 1.03–1.21, *P* = 0.008) analyses. CD4 RA expression on TD CD4^+^ cells was associated with an OR of 1.04 (95% CI = 1.00–1.09, *P* = 0.035, P_FDR_ = 0.050).

Nine immunophenotypes have been identified that provide protection against the development of MVP, including the expression of CD45 on CD33 bright HLA DR^+^, HLA DR on myeloid DC, CD19 on IgD^−^ CD38^−^, CD14^+^ CD16^+^ monocyte ACs, CD86^+^ plasmacytoid DCs ACs, HLA DR on B cells, Sw mem, SSC-A on B cells, and CD45 on CD14^+^ monocytes (Figure 3).

The OR for CD45 on CD33^br^ HLA DR^+^ in relation to MVP risk was estimated at 0.95 (95% CI = 0.90–1.00, *P* = 0.035, P_FDR_ = 0.047) using the IVW method. Similarly, the OR for HLA DR on myeloid DCs and MVP risk was calculated to be 0.92 (95% CI = 0.86–1.00, *P* = 0.036, P_FDR_ = 0.046) using the IVW method. For CD19 on IgD^−^ CD38^−^ cells, the OR was found to be 0.92 (95% CI = 0.85–1.00, *P* = 0.042, P_FDR_ = 0.044), and for CD14^+^ CD16^+^ monocyte antigen-presenting cells (AC), the OR was 0.90 (95% CI = 0.83–0.98, *P* = 0.014, P_FDR_ = 0.066), both estimated with the IVW method. The OR for CD86^+^ plasmacytoid DCs ACs on MVP risk was determined to be 0.90 (95% CI = 0.82–0.99, *P* = 0.023, P_FDR_ = 0.058) using the IVW method. Similar results were observed by using two more methods: Weighted mode (OR = 1.16, 95% CI = 1.00–1.30, *P* = 0.018) and WM (OR = 0.85, 95% CI = 0.75–0.96, *P* = 0.019). For HLA DR on B cells, the OR was 0.89 (95% CI = 0.83–0.95, *P* = 0.001, P_FDR_ = 0.046), and for switched memory AC, the OR was 0.87 (95% CI = 0.77–0.98, *P* = 0.026, P_FDR_ = 0.055), both estimated with the IVW method. The OR for SSC-A on B cells was 0.87 (95% CI = 0.77–0.99, *P* = 0.033, P_FDR_ = 0.051), and for CD45 on CD14^+^ monocytes, it was 0.86 (95% CI = 0.75–0.99, *P* = 0.031, P_FDR_ = 0.051), also estimated using the IVW method.

**Figure 4:**
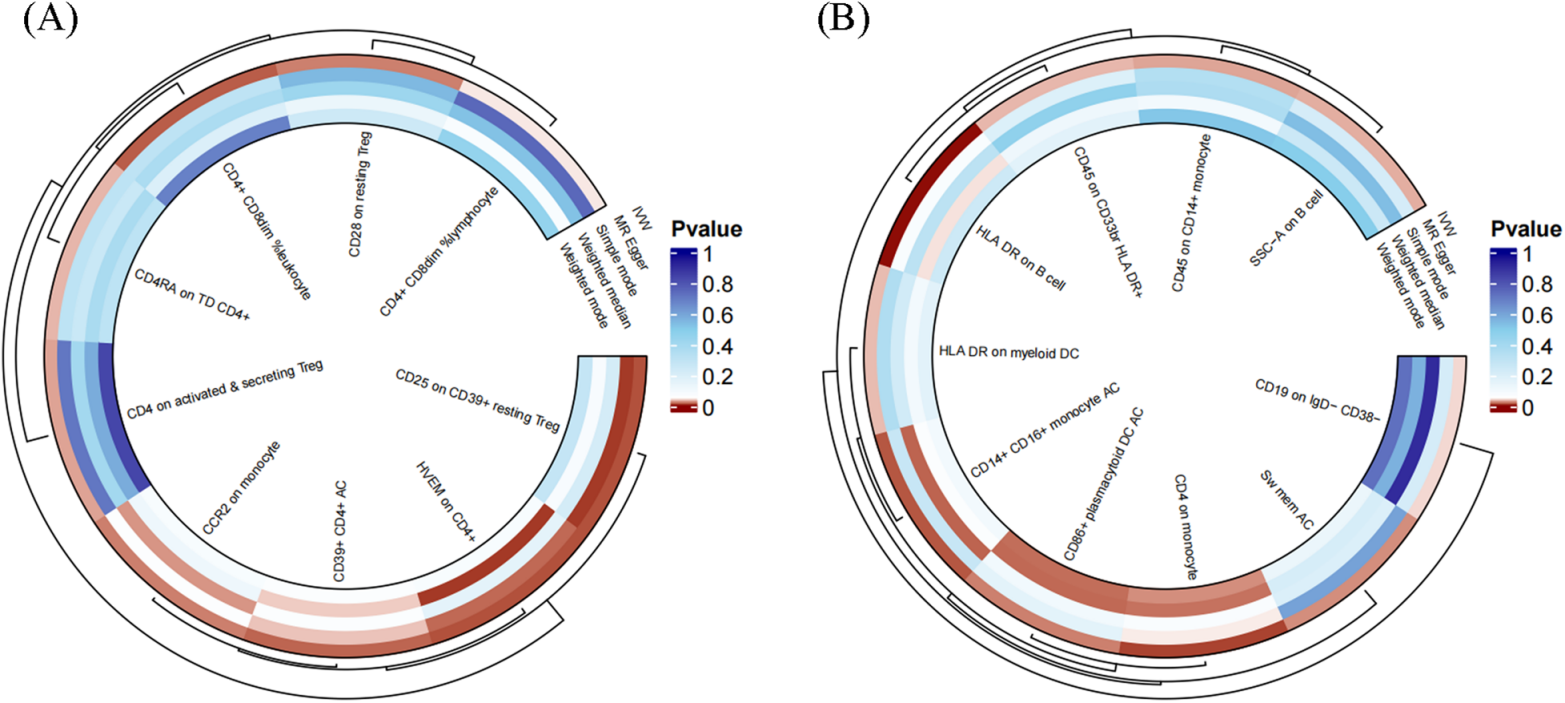
The MR analysis of immunophenotypes to MVP was shown by a circos plot. **(A)**, The circos plot displayed the results of MR analysis about nine immunophenotypes promoting MVP, including inverse variance weighted, MR Egger, simple mode, weighted median, and weighted mode. **(B)**, The circos plot displayed the results of sensitivity analyses about nine immunophenotypes protecting MVP, including inverse variance weighted, MR Egger, simple mode, weighted median, and weighted mode.

To facilitate the presentation of MR analysis for these eighteen immunophenotypes, we used a circos plot for data visualization (Figure 4 and Supplementary Table S1).

Additionally, the study utilized scatter plots, forest plots, funnel plots, and leave-one-out plots to demonstrate the robustness of these findings (Supplementary Figures S1 – S4). The assessment of diversity and horizontal pleiotropy among the eighteen immune cell types indicated no signs of horizontal pleiotropy (Table 2). However, significant heterogeneity was observed in CD4+ CD8^dim^% lymphocytes and leukocytes, whereas other immunophenotypes exhibited no significant heterogeneity (Table 2).

**Table 2:** Tests for pleiotropy and heterogeneity between immune cells and MVP.

Panel	Exposure	Number of SNPs	MR_Egger Regression		Heterogeneity		
			Intercept	P_intercept	Method	Q	Q_pval
TBNK	HLA DR on B cell	15	0.00	0.93	MR Egger	16.43	0.23
					IVW	16.44	0.29
	SSC-A on B cell	9	0.02	0.61	MR Egger	5.33	0.62
					IVW	5.61	0.69
	CD45 on CD14 + monocyte	8	0.00	0.97	MR Egger	7.13	0.31
					IVW	7.14	0.41
	CD4+ CD8dim%lymphocyte	16	0.02	0.51	MR Egger	28.52	0.01
					IVW	29.43	0.01
	CD4+ CD8dim%leukocyte	12	−0.03	0.61	MR Egger	21.53	0.02
					IVW	22.11	0.02
Treg	CD25 on CD39 + resting Treg	13	−0.04	0.06	MR Egger	9.05	0.62
					IVW	13.51	0.33
	CD28 on resting Treg	5	0.02	0.57	MR Egger	3.16	0.37
					IVW	3.58	0.47
	CD39+ CD4+ AC	23	−0.01	0.36	MR Egger	30.17	0.09
					IVW	31.45	0.09
	CD4 on activated & secreting Treg	22	0.02	0.07	MR Egger	19.39	0.50
					IVW	22.93	0.35
Maturation stages of T cell	HVEM on CD4+	15	−0.03	0.12	MR Egger	11.46	0.57
					IVW	14.24	0.43
	CD4RA on TD CD4+	23	−0.01	0.58	MR Egger	19.91	0.53
					IVW	20.77	0.53
B cell	Sw mem AC	15	−0.01	0.58	MR Egger	19.32	0.11
					IVW	19.81	0.14
	CD19 on IgD- CD38-	18	0.00	0.86	MR Egger	18.88	0.27
					IVW	18.92	0.33
Monocyte	CD14+ CD16 + monocyte AC	16	−0.01	0.45	MR Egger	19.03	0.16
					IVW	19.87	0.18
	CCR2 on monocyte	14	−0.01	0.58	MR Egger	15.55	0.21
					IVW	15.97	0.25
cDC	HLA DR on myeloid DC	8	0.00	0.85	MR Egger	5.89	0.44
					IVW	5.92	0.55
	CD86 + plasmacytoid DC AC	16	0.00	0.89	MR Egger	17.33	0.24
					IVW	17.36	0.30
Myeloid cell	CD45 on CD33br HLA DR +	12	0.00	0.86	MR Egger	8.33	0.60
					IVW	8.36	0.68

MVP, mitral valve prolapse; SNPs, Single-nucleotide polymorphisms; MR, Mendelian randomization; IVW, Inverse variance weighted; TBNK, T cells, B cells, natural killer cells; AC, absolute cell; Sw mem, switched memory; DC, dendritic cell; HLA, Human Leucocyte Antigen; TD, terminally differentiated; br, bright.

### 3.2 Exploration of the causal effect of MVP on immunophenotypes

**Figure 5:**
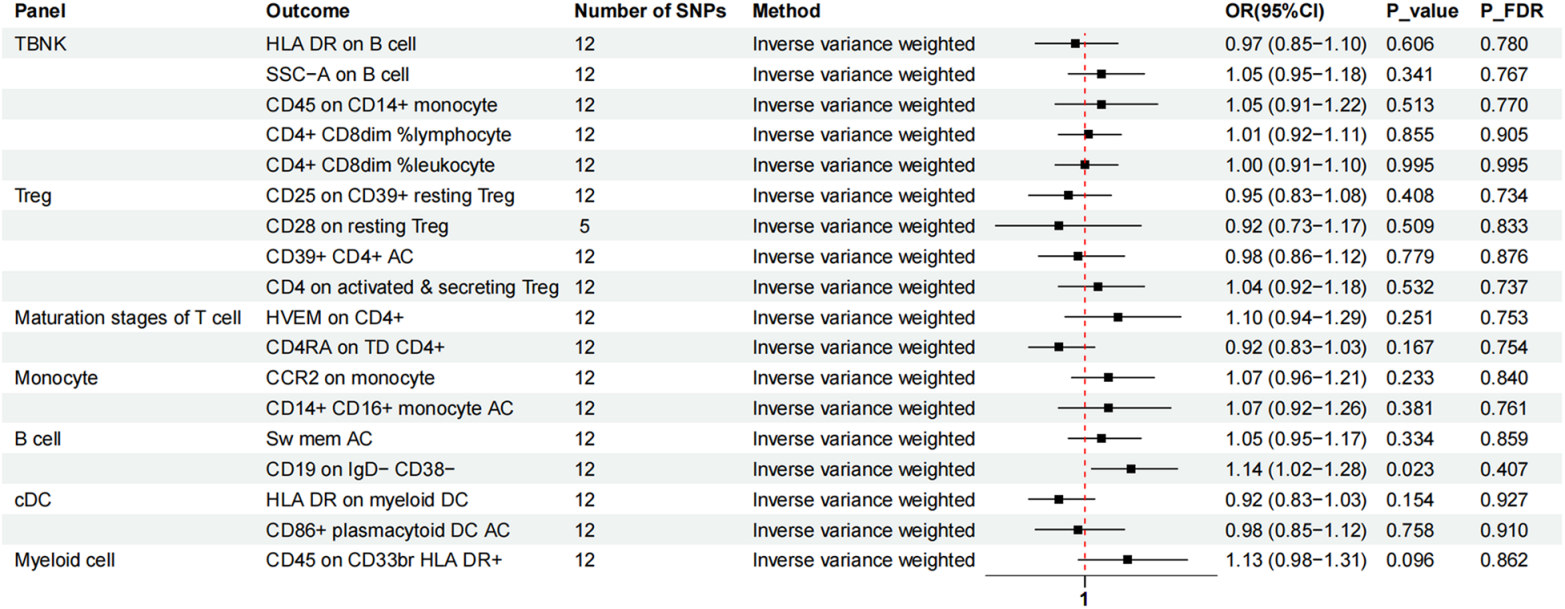
The forest plot showed the causal association between MVP and immunophenotypes by the IVW method. TBNK, T cells, B cells, natural killer cells; AC, absolute cell; Sw mem, switched memory; DC, dendritic cell; HLA, human leucocyte antigen; br, bright; TD, terminally differentiated.

We again used IVW as the primary result of reverse MR on the previous eighteen immunophenotypes. After FDR adjustment significance of 0.10, MVP had no causal relationship for any immune cell phenotype (Figure 5).

## 4 Discussion

The research examined the causal relationship between 731 immunophenotypes and MVP using publicly available genetic data. Currently, limited studies focus on the association between immunophenotypes and MVP. To the best of our knowledge, this is the inaugural study to assess the connection between immunophenotypes and MVP through MR. Our results revealed a significant causal relationship between MVP and 18 immunophenotypes (PFDR < 0.1). Conversely, reverse MR analysis showed that MVP does not causally influence these 18 crucial immunophenotypes, suggesting that MVP does not interact with immunophenotypes during the disease progression. This finding preliminarily ruled out the possibility that MVP affected these 18 immunophenotypes during the disease process and reverse causation.

The findings highlighted the role of T cells (CD4+ CD8dim% lymphocyte, CD4+ CD8dim% leukocyte, HVEM on CD4^+^, and CD4 RA on TD CD4^+^) in the development of MVP. CD4^+^ T cells are implicated in inflammation and fibrosis during cardiac remodeling (30). In dogs with myxomatous mitral valve disease (MMVD), an increase in CD4^+^ CD3^+^ T cell counts was noted in severely affected animals, along with elevated levels of inflammatory cytokines [tumor necrosis actor-α, interleukin (IL)-1β, and IL-6] (31). Additionally, CD4^+^ T cells and the TGF*β*1/MAPK pathway have been identified as contributors to valvular hyperplasia and fibrosis in individuals with rheumatic heart disease (32). Adult T-cell leukemia/lymphoma (ATLL), a severe mature T-cell tumor, has been observed to involve the mitral valve, showing CD4^+^ T-cell infiltration in chronic cases (33, 34). TNFSF14, also known as LIGHT or CD258, is crucial in immune responses, primarily through its interaction with the lymphotoxin *β* receptor (LT*β*R) and the HVEM (or TNFRSF14), both prevalent in T lymphocytes and other immune cells (35). The association between MVP and HVEM has yet to be explored. However, HVEM is known to enhance TGF-*β* expression and has a pro-inflammatory and pro-fibrotic role in atrial fibrillation (36), and is associated with poor prognosis in patients with heart failure (37).

Despite the anti-inflammatory role of regulatory T cells (Tregs), our findings indicate an association between Tregs (CD25 on CD39^+^ resting Tregs, CD28 on resting Tregs, CD4 on activated and secreting Tregs, CD39^+^ CD4^+^ AC) and an increased risk of MVP. Notably, Treg levels were higher in dogs with MMVD than in older, healthy dogs (31). Similar to Marfan syndrome, Loeys-Dietz syndrome is an autosomal dominant disorder caused by mutations in the gene encoding the TGF-*β* receptor subunit, which is also a cause of mitral valve prolapse. The dysregulation of TGF-*β* and the promotion of Smad2 and Smad3 protein phosphorylation and nuclear translocation are linked to modifications in the frequencies and functions of Tregs and treatment with losartan rescued this phenotype (38). Tregs were also found to be elevated in aortic stenosis patients, and following aortic valve intervention, the total number of Tregs decreased (39). Aligning with our analysis, these studies insinuate potential involvement of Tregs in MVP pathogenesis.

CCR2^+^ monocytes are involved in the myxomatous valve disease (15, 16). Notably, Na Xu et al. have proved targeting CCR2 may be a potential medical therapy in both the early and late phases of MVD in MFS by using C-C chemokine receptor type 2 genetic knockout mice and C-C chemokine receptor type 2 antagonist RS504393 (40).

The MR analysis suggested that HLA DR^+^ expression on myeloid cells, DCs, and B cells might offer protection against MVP. Decreased HLA DR expression on monocytes and B cells post-cardiac surgery indicates immune suppression (41). An inverse relationship between HLA DR5 and rheumatic valvular heart disease, as reported by Ozkan et al., suggests a protective role (42).

The risk of MVP may be reduced by memory B cells. Autoimmune retinopathy (AIR) is linked to autoimmunity, with primary abnormalities found in B lymphocyte differentiation. Elena Stansky and colleagues showed that patients with AIR had an increased frequency of naive B cells, while switch memory B cells and plasmablasts were significantly decreased (43). Elevated levels of (un)switched and unswitched memory B cells have been correlated with improved outcomes after carotid artery endarterectomy, suggesting that specific B-cell subgroups could act as predictors and preventers of cardiovascular events in patients with atherosclerosis (44).

Compared with CCR2^+^ monocytes, CD16^+^ monocytes, alongside CD86^+^ plasmacytoid DCs (pDC), may offer a protective effect on MVP. In patients with rheumatoid arthritis, the induction of CD14^+^ CD16^+^ monocytes and the differentiation of M2 macrophages, which play an anti-inflammatory role, were facilitated by platelet-derived TGF-*β* and monocyte-derived IL-6 (45). Moreover, non-classical monocytes may contribute to anti-atherosclerosis efforts and promote myocardial injury healing (46). Recent research has shown that in patients with heart failure before cardiac resynchronization therapy (CRT), there is a lower frequency of pDC, which increases CD86 expression post-CRT. This study observed that classical monocyte levels (associated with pro-inflammatory effects) decreased, whereas intermediate monocytes (beneficial and anti-inflammatory) and nonclassical monocytes (important for wound healing) increased post-CRT. It suggests that pDC and monocytes play roles in cardiac remodeling and respond positively to CRT (47).

This study utilized data from large GWAS cohorts to conduct a highly statistically efficient two-sample MR analysis. Various MR analysis techniques were applied to infer causality based on genetic IVs, with the results showing robustness unaffected by horizontal pleiotropy. However, several limitations were acknowledged. First, horizontal pleiotropy could not be fully assessed through multiple sensitivity analyses. Heterogeneity was observed in two immunophenotypes, attributed to differences in populations and experimental conditions. The generalizability of the results is limited to European descent, restricting applicability to other ethnic groups. Additionally, the lack of clinical and basic experiments to support the data's reliability highlights the need for various types of experiments to confirm these conclusions. Lastly, a broader threshold for data analysis was applied, potentially increasing false positives but allowing a more comprehensive evaluation of the robust correlation between the immune profile and MVP.

## 5 Conclusion

This MR study is believed to be the first to investigate the causal relationship between immunophenotypes and MVP, significantly reducing reverse causality and other confounding factors. Thus, it may offer new insights into basic and clinical research of MVP from an immunity standpoint.

## Data Availability

Publicly available datasets were analyzed in this study. This data can be found here: https://cvd.hugeamp.org/; https://www.ebi.ac.uk/gwas/home.
