# Supplementary material for: Causal relationship between immunophenotypes and mitral valve prolapse: a bidirectional Mendelian randomization study
Source: Front Cardiovasc Med. 2024 Oct 3;11:1404284. doi: 10.3389/fcvm.2024.1404284 (PMC11484250; doi:10.3389/fcvm.2024.1404284)
Supplement: Supplementary file 1 [file Datasheet1.pdf]

Supplementary Figures

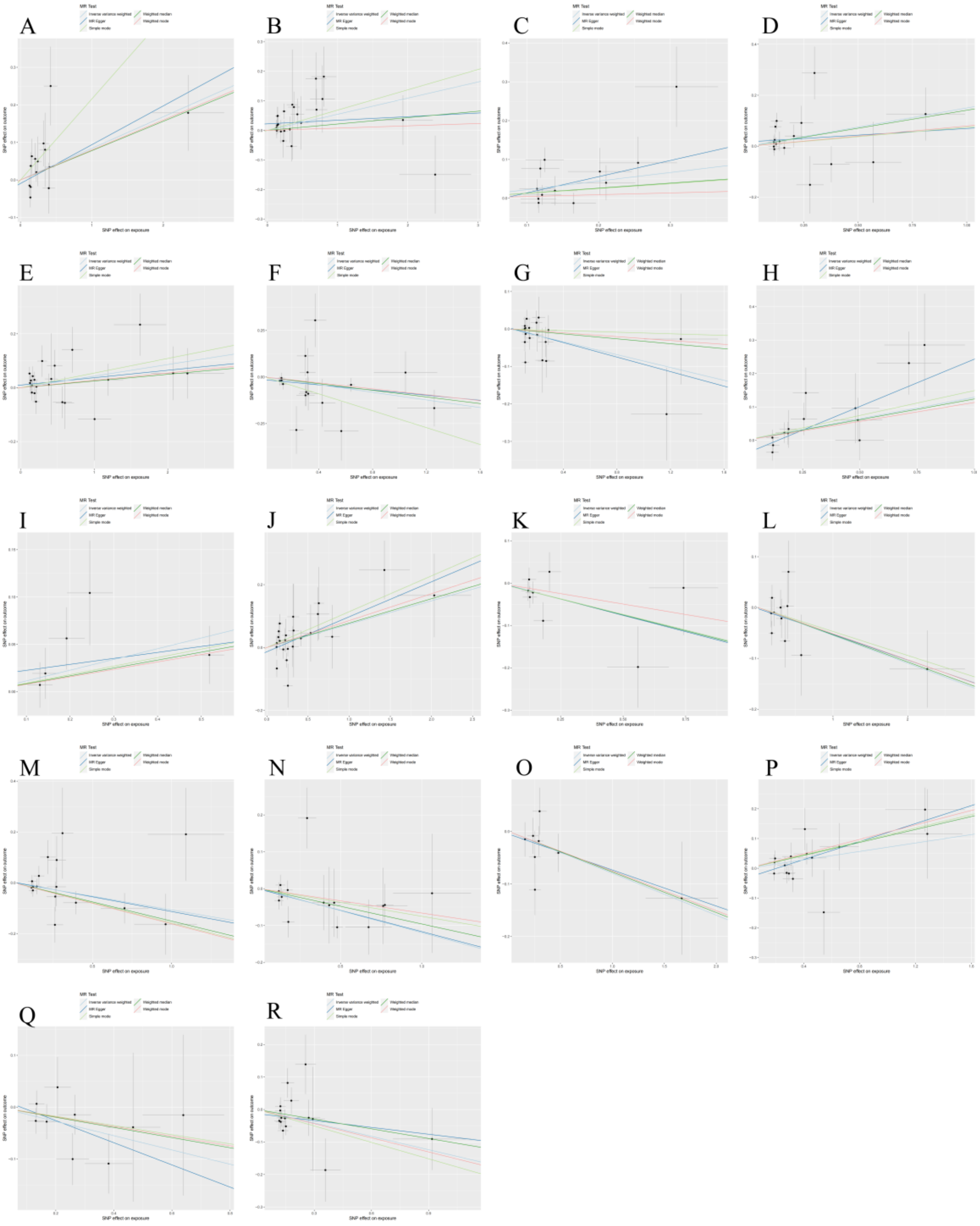

Supplementary Figure 1. Causal effects of immune cells on MVP. (A). Scatter plot between CCR2 on monocyte and MVP risk; (B). Scatter plot between CD4 on activated & secreting Treg and MVP risk; (C). Scatter plot between CD4+ CD8dim %leukocyte and MVP risk; (D). Scatter plot between CD4+ CD8dim %lymphocyte and MVP risk; (E). Scatter plot between CD4RA on TD CD4+ and MVP risk; (F). Scatter plot between CD14+ CD16+ monocyte AC and MVP risk; (G). Scatter plot between CD19 on IgD- CD38- and MVP risk; (H). Scatter plot between CD25 on CD39+ resting Treg and MVP risk; (I). Scatter plot between

CD28 on resting Treg and MVP risk; (J). Scatter plot between CD39+ CD4+ AC and MVP risk; (K). Scatter plot between CD45 on CD14+ monocyte and MVP risk;(L). Scatter plot between CD45 on CD33br HLA DR+ and MVP risk; (M). Scatter plot between CD86+ plasmacytoid DC AC and MVP risk; (N). Scatter plot between HLA DR on B cell and MVP risk; (O). Scatter plot between HLA DR on myeloid DC and MVP risk; (P). Scatter plot between HVEM on CD4+ and MVP risk; (Q). Scatter plot between SSC-A on B cell and MVP risk; (R). Scatter plot between Sw mem %B cell and MVP risk.

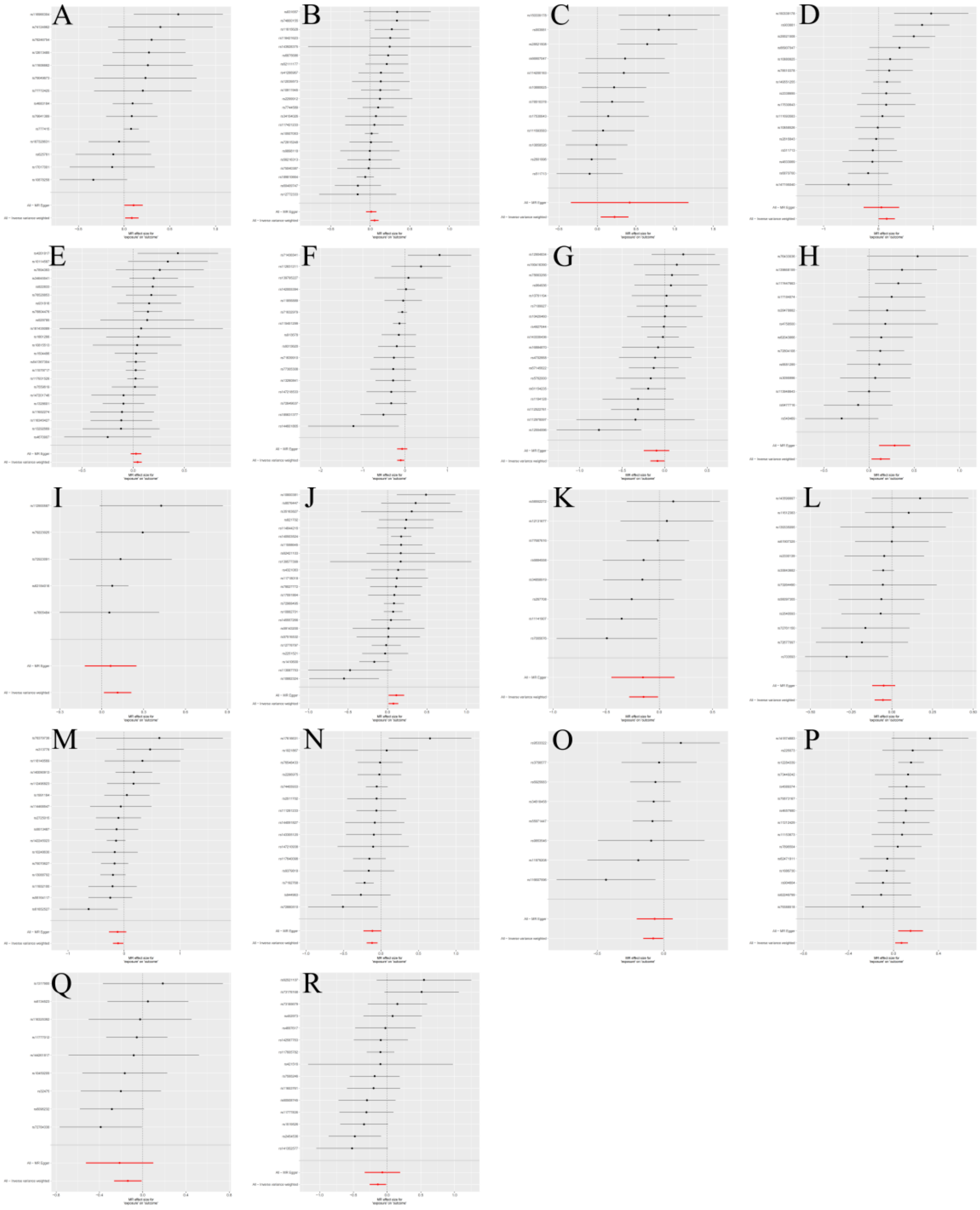

Supplementary Figure 2. Forest plots between immune cells on MVP. (A). Forest plot between CCR2 on monocyte and MVP risk; (B). Forest plot between CD4 on activated & secreting Treg and MVP risk; (C). Forest plot between CD4+ CD8dim %leukocyte and MVP risk; (D). Forest plot between CD4+ CD8dim %lymphocyte and MVP risk; (E). Forest plot between CD4RA on TD CD4+ and MVP risk; (F). Forest plot between CD14+ CD16+ monocyte AC and MVP risk; (G). Forest plot between CD19 on IgD- CD38- and MVP risk; (H). Forest plot between CD25 on CD39+ resting Treg and MVP risk; (I). Forest plot between CD28 on resting Treg and MVP risk; (J). Forest plot between CD39+ CD4+ AC and MVP

risk; (K). Forest plot between CD45 on CD14+ monocyte and MVP risk;(L). Forest plot between CD45 on CD33br HLA DR+ and MVP risk; (M). Forest plot between CD86+ plasmacytoid DC AC and MVP risk; (N). Forest plot between HLA DR on B cell and MVP risk; (O). Forest plot between HLA DR on myeloid DC and MVP risk; (P). Forest plot between HVEM on CD4+ and MVP risk; (Q). Forest plot between SSC-A on B cell and MVP risk; (R). Forest plot between Sw mem %B cell and MVP risk.

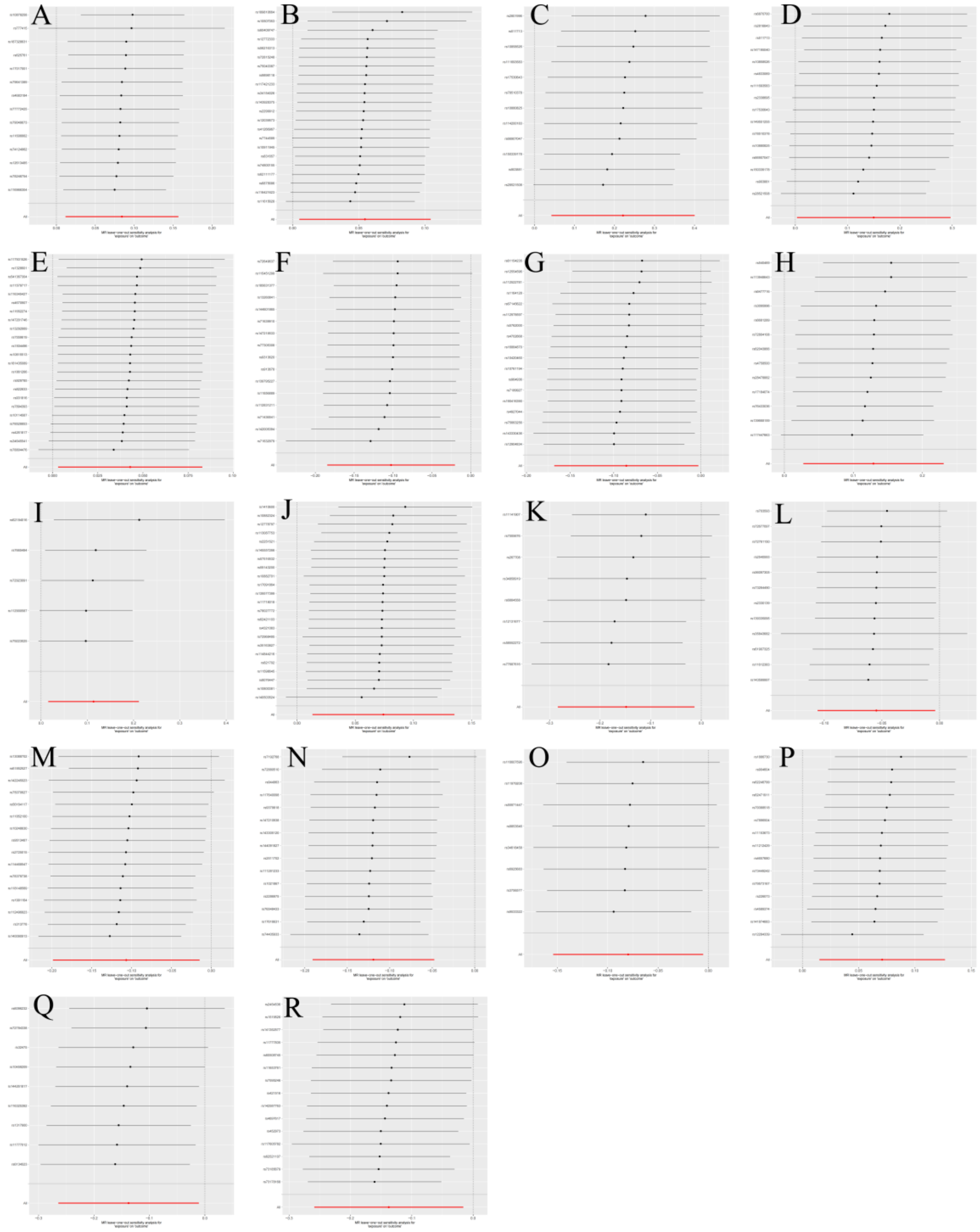

Supplementary Figure 3. Leave-one-out plots for the causal association between immune cells and MVP. (A). Leave-one-out plot between CCR2 on monocyte and MVP risk; (B). Leave-one-out plot between CD4 on activated & secreting Treg and MVP risk; (C). Leave-one-out plot between CD4+ CD8dim %leukocyte and MVP risk; (D). Leave-one-out plot between CD4+ CD8dim %lymphocyte and MVP risk; (E). Leave-one-out plot between CD4RA on TD CD4+ and MVP risk; (F). Leave-one-out plot between CD14+ CD16+ monocyte AC and MVP risk; (G). Leave-one-out plot between CD19 on IgD- CD38- and MVP risk; (H). Leave-one-out plot between CD25 on CD39+ resting Treg and MVP risk; (I). Leave-one-out plot between CD28 on resting Treg and MVP risk; (J). Leave-one-out plot between

CD39+ CD4+ AC and MVP risk; (K). Leave-one-out plot between CD45 on CD14+ monocyte and MVP risk;(L). Leave-one-out plot between CD45 on CD33br HLA DR+ and MVP risk; (M). Leave-one-out plot between CD86+ plasmacytoid DC AC and MVP risk; (N). Leave-one-out plot between HLA DR on B cell and MVP risk; (O). Leave-one-out plot between HLA DR on myeloid DC and MVP risk; (P). Leave-one-out plot between HVEM on CD4+ and MVP risk; (Q). Leave-one-out plot between SSC-A on B cell and MVP risk; (R). Leave-one-out plot between Sw mem %B cell and MVP risk.

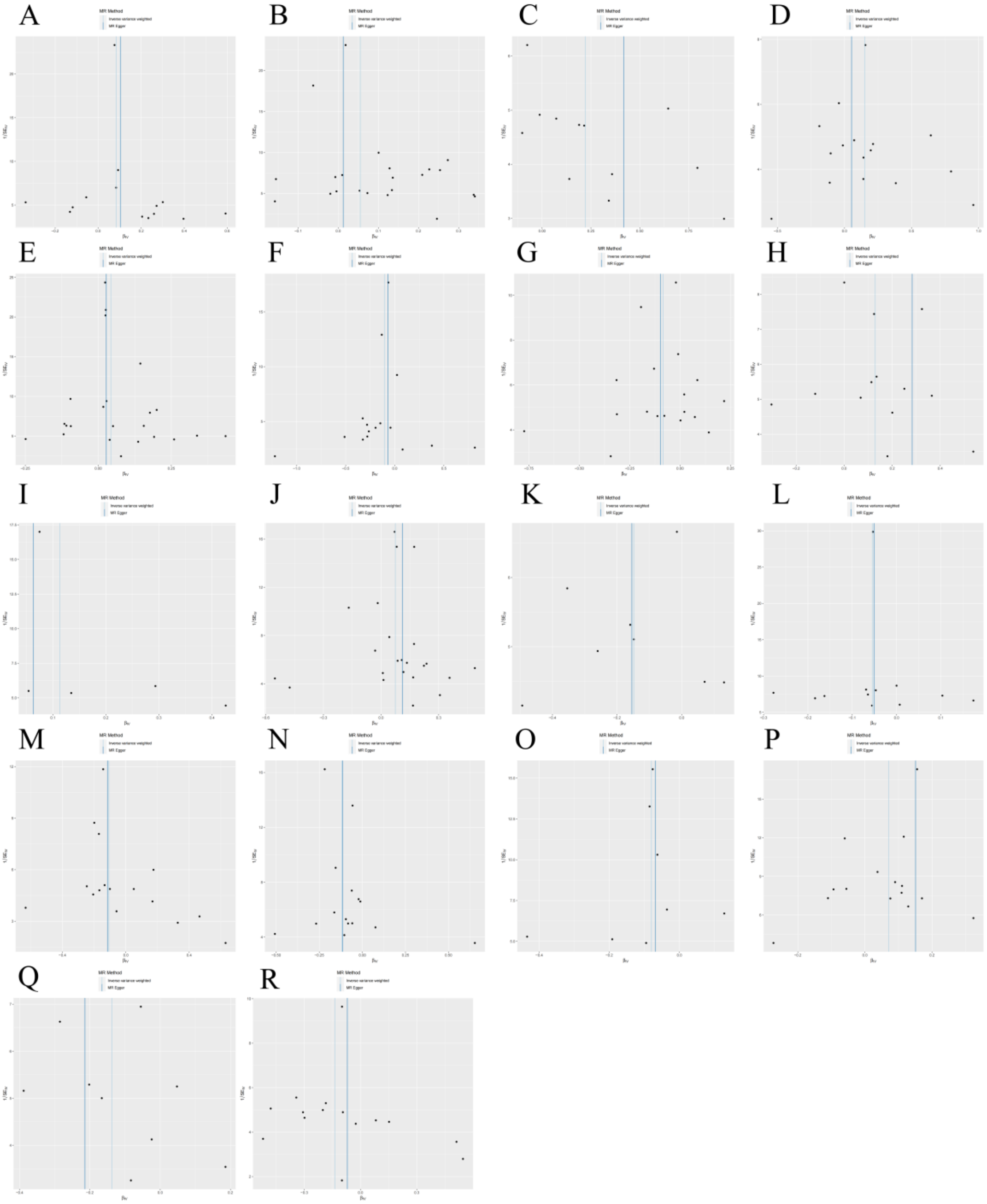

Supplementary Figure 4. Funnel plots for the causal association between immune cells and MVP. (A). Funnel plot between CCR2 on monocyte and MVP risk; (B). Funnel plot between CD4 on activated & secreting Treg and MVP risk; (C). Funnel plot between CD4+ CD8dim %leukocyte and MVP risk; (D). Funnel plot between CD4+ CD8dim %lymphocyte and MVP risk; (E). Funnel plot between CD4RA on TD CD4+ and MVP risk; (F). Funnel plot between CD14+ CD16+ monocyte AC and MVP risk; (G). Funnel plot between CD19 on IgD- CD38- and MVP risk; (H). Funnel plot between CD25 on CD39+ resting Treg and MVP risk; (I). Funnel plot between CD28 on resting Treg and MVP risk; (J). Funnel plot between CD39+ CD4+ AC and MVP risk; (K). Funnel plot between CD45 on CD14+ monocyte and MVP risk;(L).

Funnel plot between CD45 on CD33br HLA DR+ and MVP risk; (M). Funnel plot between CD86+ plasmacytoid DC AC and MVP risk; (N). Funnel plot between HLA DR on B cell and MVP risk; (O). Funnel plot between HLA DR on myeloid DC and MVP risk; (P). Funnel plot between HVEM on CD4+ and MVP risk; (Q). Funnel plot between SSC-A on B cell and MVP risk; (R). Funnel plot between Sw mem %B cell and MVP risk.
